# *Allium*-Derived Compound Propyl Propane Thiosulfonate (PTSO) Reduces Vibrio Populations and Increases Body Weight of European Seabass (*Dicentrarchus labrax*) Juveniles

**DOI:** 10.3390/antibiotics12010134

**Published:** 2023-01-10

**Authors:** Miguel Rabelo-Ruiz, Juan Manuel Peralta-Sánchez, Antonio Manuel Martín-Platero, Ana J. Ruiz, María del Mar Agraso, Laura Bermúdez, Juan José Ariza, Alberto Baños, Eva Valdivia, Manuel Martínez-Bueno

**Affiliations:** 1 Departament of Microbiology, University of Granada, Avda. Fuentenueva, s/n, 18071 Granada, Spain; 2 Aquaculture Technology Centre of Andalusia, CTAQUA. Muelle Comercial s/n, El puerto de Santa María, 11500 Cádiz, Spain; 3 Departament of Microbiology and Biotecnology, DMC Research Center, Camino de Jayena s/n, 18620 Granada, Spain; 4 Institute of Biotecnology, University of Granada, 18071 Granada, Spain

**Keywords:** *Allium*-derived phytobiotic, body weight, European seabass (*Dicentrarchus labrax*) juveniles, gut microbiota, propyl propane thiosulfonate (PTSO)

## Abstract

The global demand for fish products is continuously increasing as the population grows, and aquaculture plays an important role in supplying this demand. However, industrial antibiotic misuse has contributed to the spread of antimicrobial resistance among pathogenic bacteria, therefore, several antibiotic alternatives have been proposed. In this study, we have analyzed the effects of *Allium*-derived propyl propane thiosulfonate (PTSO) in European seabass juveniles’ growth and performance. These effects were tested by measuring the body weight and analyzing the gut microbiome of fish after 89 days of feeding trial. The relative abundance of potentially pathogenic *Vibrio* in the foregut and hindgut of supplemented fish decreased, while *Pseudomonas* and *Kocuria* increased significantly. Alpha diversity indices significantly decreased in both gut regions of fish fed with *Allium-*derived PTSO supplemented diet, as well as between bacterial community composition. These results may indicate a positive effect of the supplementation in the diet with *Allium*-derived PTSO, reducing potentially pathogenic *Vibrio* and increasing body weight at the end of the experiment (89 days). However, this supplementation with *Allium-*derived PTSO produces changes in the diversity and composition of microbial communities, so further experiments would be necessary to explore bacterial community composition and health relationship.

## 1. Introduction

World population has increased exponentially in the last years, and it is expected to continue growing in the coming years, reaching 9.7 billion in the year 2050 and almost 11 billion people worldwide in the year 2100 [1]. This increase in world population implies an increase in food demand, which can be partially covered by aquaculture products, given the high impact of land-based animal production and the stagnation of wild fishery catches [2]. Currently, this industry plays an important role in supplying the world food demand and protein source, with a global aquaculture production of 82 million tons in 2018 and an economic value of USD $250 billion [3]. However, economic profits in the industry are affected by fish diseases caused by several pathogenic bacteria such as *Aeromonas*, *Vibrio,* or *Photobacterium* [4]. Infections of these pathogenic bacteria are treated by high doses of antibiotics, given the high fish stocking densities and the impossibility of individual treatment [5]. Furthermore, in the aquaculture industry, antibiotics have been used as growth promoters (antibiotic growth promoters, AGP) for several years, showing an improvement in feed efficiency and growth performance in different fish species [6,7]. However, the extensive use of antibiotics for growth-promoting and therapeutic purposes in aquaculture systems has increased the antibiotic resistance of pathogenic bacteria [8]. Therefore, a worldwide effort is necessary to reduce and rationalize the use of antibiotics in livestock and aquaculture. For this reason, the use of antibiotic growth promoters (AGPs) in animal feed was banned by the European Union in 2006 [9] and by other countries in the following years [10,11].

Several feed additives have been proposed as alternatives to AGPs in the aquaculture industry. The most promising alternatives include enzymes, bacteriophages, probiotics, prebiotics, and phytobiotics [12,13,14]. Phytobiotics are defined as plant-derived bioactive compounds supplemented in the diet to improve animal productivity [15]. Phytobiotics are known to have antimicrobial activity against pathogenic bacteria and can act as prebiotics, facilitating a continuous supply of specific substrates for intestinal microbiota or minimizing the risk of pathogenic bacteria development [16]. These products also act as stimulant of saliva and bile secretion, which helps to increase productive parameters [17]. Many potential herbal plants have been identified and used in aquaculture for improvement of fish health, including more than 60 different medicinal plant species [18,19].

*Allium* species, mainly garlic (*Allium sativum*) and onion (*Allium cepa*), produce a wide variety of bioactive compounds with antifungal, antimicrobial, and antioxidant activity [20]. Dietary supplementation of these compounds has shown promising results, improving the health and productive parameters of goats, cattle, pigs and poultry [21]. The use of *A. cepa* extract in cattle produced no changes in milk attributes [22], while in goats, *A. sativum* oil showed a beneficial effect in the milks’ fatty acid profile [23]. The inclusion of *Allium* in growing finishing pigs showed a reduction of *Salmonella*, an increase in *Lactobacillus* and acid levels in feces [24], and an increased growth performance [25]. In the poultry industry, *Allium* supplementation in laying hens improved health status, intestinal microbiota, and increased egg size and weight [26,27]; it also increased growth performance, immunity, and antioxidant status of broiler chickens [28,29]. Application of *Allium* species in fish farming has become popular for promoting growth, improving the activity of defense systems, and protecting against diseases caused by pathogenic bacteria [21,30]. The inclusion of onion (*A. cepa*) powder in the diet of beluga juveniles (*Huso huso*) improved growth performance, immune function, and blood parameters [31]. Regarding the dietary supplementation of garlic (*A. sativum*) extract, it has been proven that it promotes growth, enhances the immune system, and improves the control of pathogens [30,32]. Inclusion of garlic in diet showed an increase in weight gain and growth rate of rainbow trout (*Oncorhynchus mykiss*) [33], an improvement of food digestibility and biochemical and immunohematological effects of Eurasian perch (*Perca fluviatilis*) juveniles [34], as well as an increase in the immune parameters of skin mucus of guppy fish (*Poecilia reticulata*) [35]. Furthermore, dietary inclusion of garlic has demonstrated its ability to control pathogens of host, showing antimicrobial activity against fungi and bacteria, including *Pseudomonas fluorescens* or *Vibrio anguillarum* [30,32].

The activity of these plant compounds has been related to secondary metabolites, volatile organosulfur compounds such as ajoene, allicin, isoalliin, methiin, propiin, propyl propane thiosulfonate (PTSO), and propyl propane thiosulfinate (PTS) [30,36]. PTS and PTSO (Supplementary Figure S1) have shown antibacterial, antifungal [37,38], and anticoccidial activity [39]. Furthermore, PTSO showed beneficial effects on intestinal health in several animal species [36] and changes in gut microbiota and growth performance of different livestock animals, such as mice, broiler chickens, laying hens, and pigs [24,27,40,41,42,43,44]. In addition, recent studies using experimental animals have shown that PTSO is a toxicologically safe compound [45]. However, the potential effects of *Allium-*derived PTSO on intestinal microbiota and body weight of European seabass juveniles has not yet been explored.

Hence, our aim in this study was to evaluate the effects of *Allium*-derived PTSO on the European seabass (*D. labrax*) juveniles’ growth performance, as well as its foregut and hindgut microbiota via high-throughput sequencing of the V6-V8 region of 16S rRNA gene. As described below, this approach shows that the inclusion of this *Allium*-based product increases fish growth performance and induces changes in the gut microbiota after 89 days of feeding trial, including the reduction of potential pathogens such as *Vibrio* populations.

## 2. Results

### 2.1. Effect of Feeding Diet on European Seabass Juvenile Growth Performance

**Figure 1:**
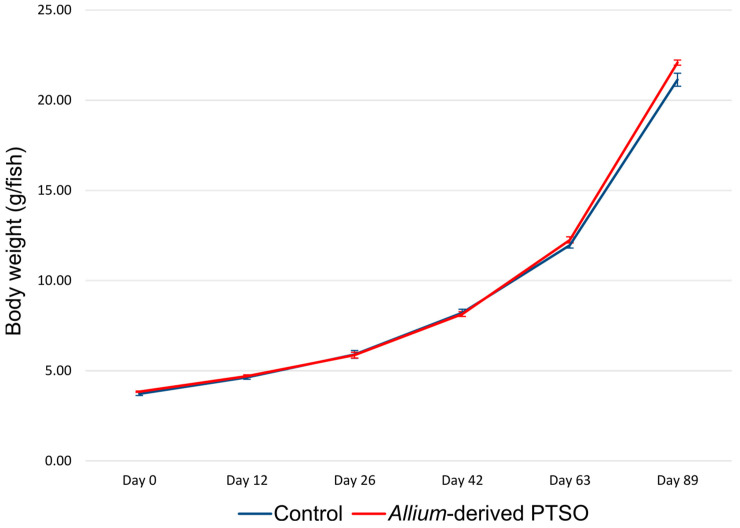
Evolution of growth performance of European seabass (*D. labrax*) juveniles fed with control diet or supplemented with *Allium*-derived PTSO along the feeding trial. Error bars show standard error.

No differences appeared in the initial body weight between the fish fed with the control or *Allium*-derived PSTO supplemented diet (Table 1, Figure 1). European seabass juveniles supplemented with *Allium-*derived PTSO showed an increase in body weight at the end of the feeding trial (Day 89) (Table 1, Figure 1). However, no differences in body weight appeared between fish fed with a control diet or *Allium*-derived PTSO along the experiment, showing similar body weight at days 12, 26, 42, and 63 (Table 1, Figure 1).

### 2.2. Bacterial Community Composition

**Figure 2:**
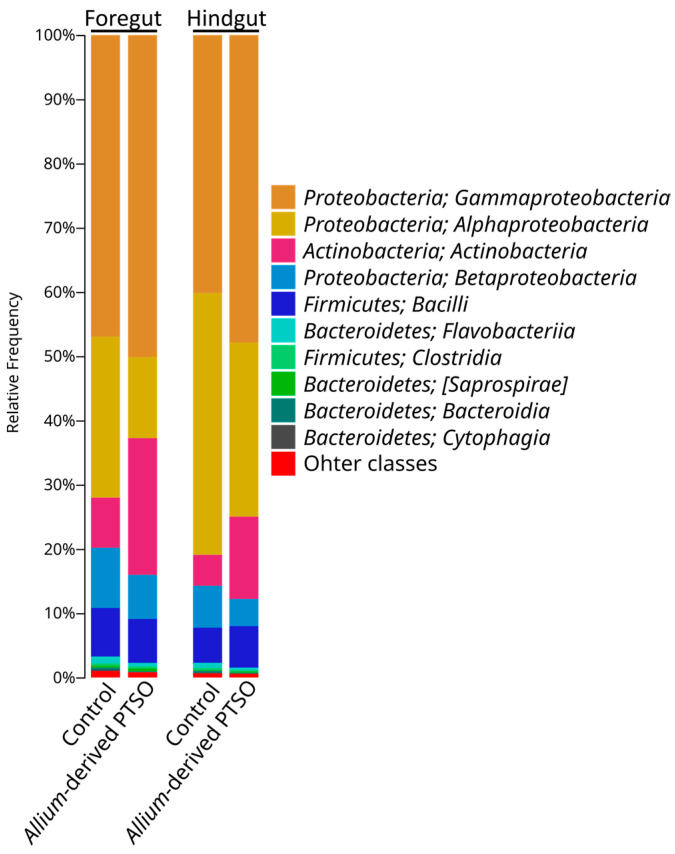
Microbial composition at the class level of juvenile European seabass gut microbiota group by experimental diet (control and *Allium*-derived PTSO). Classes in the legend are sorted from most abundant to lowest abundant.

**Figure 3:**
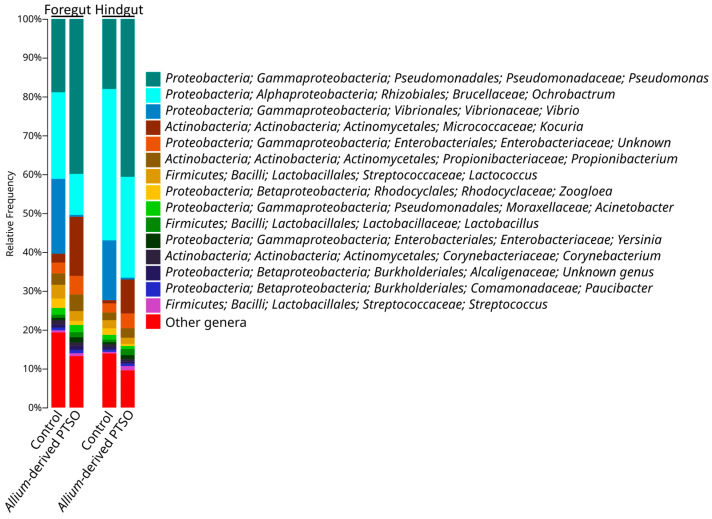
Microbial composition at the genus level of juvenile European seabass gut microbiota group by diet (control and *Allium-*derived PTSO). Genera in the legend are sorted from most abundant to lowest abundant.

**Figure 4:**
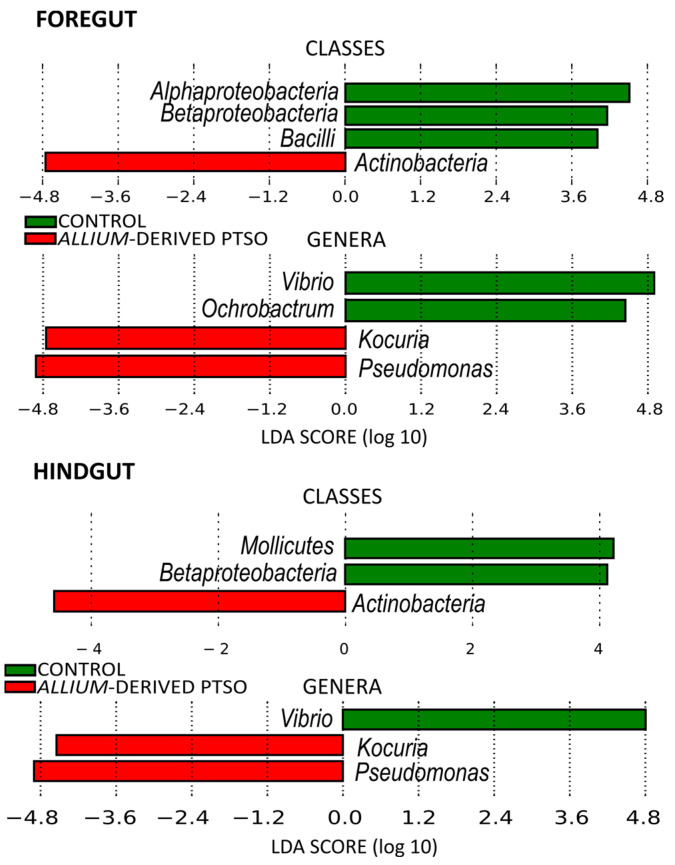
LDA Effect Size (LEfSe) analyses showing bacterial classes and genera that differ significantly between control fish and those supplemented with *Allium-*derived PTSO, in the foregut and in the hindgut of European seabass juveniles. Significant LDA Score > 4.0.

The foregut microbiota of juvenile European seabass was dominated at class level by *Gammaproteobacteria* (47%), *Alphaproteobacteria* (25%), *Betaproteobacteria* (9%), *Actinobacteria* (8%), and *Bacilli* (8%) (Figure 2). Fish supplemented with *Allium-*derived PTSO showed a significant decrease in *Alphaproteobacteria* (13%), *Betaproteobacteria* (7%), and *Bacilli* (7%), as well as an increase in *Actinobacteria* (21%) (Figure 2). At the genus level, the foregut of control fish was dominated by *Ochrobactrum* (22%), *Pseudomonas* (19%), and *Vibrio* (19%). In the foregut of *Allium*-derived PTSO fish an increase in *Pseudomonas* (40%) and *Kocuria* (15%)*,* and a decrease in *Vibrio* (<1%) and *Ochrobactrum* (11%) were observed (Figure 3 and Figure 4).

The bacterial composition of the hindgut of control fish was similar to their foregut, dominated at class level by *Alphaproteobacteria* (41%), *Gammaproteobacteria* (40%), *Betaproteobacteria* (7%), *Bacilli* (5%), and *Actinobacteria* (5%) (Figure 2). The hindgut microbiota of *Allium*-derived PTSO supplemented fish showed a significant increase in *Actinobacteria* (13%) and decrease in *Betaproteobacteria* (4%) and in the minority class *Mollicutes* (Figure 4). At the genus level, the foregut of control fish was dominated by *Ochrobactrum* (39%), *Pseudomonas* (18%), and *Vibrio* (15%). In the hindgut of *Allium*-derived PTSO supplemented fish, an increase in *Kocuria* (9% respect to 1% in control fish) and *Pseudomonas* (41%) was observed, as well as a decrease in *Vibrio* (<1%) (Figure 3 and Figure 4). 

### 2.3. Effect of Feeding Diet on Alpha and Beta Diversity

Supplementing the diet of European seabass juveniles with *Allium*-derived PTSO affected alpha diversity indices (Table 2). *Allium*-derived PTSO supplemented fish showed a reduction in alpha diversity respect to control fish. However, no differences appeared between gut region, showing both foregut and hindgut similar levels of alpha diversity. Furthermore, no differences appeared in the interaction of diet and gut region, indicating that the changes in diversity between both gut regions occurs in the same way in both feeding diets (see Diet*Gut Region interaction term in Table 2).

**Figure 5:**
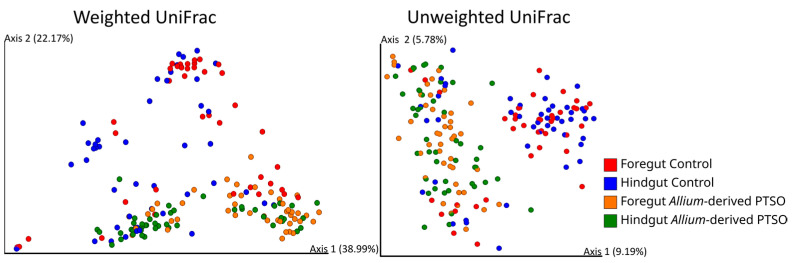
Dimensional figures showing the first two axes of principal coordinate analysis and representing bacterial communities of foregut and hindgut of juvenile European seabass fed with control diet or supplemented with *Allium-*derived PTSO using unweighted and weighted UniFrac distance matrixes. Samples are colored by gut region and experimental diet (foregut control—red; hindgut control—blue; foregut *Allium-*derived PTSO—yellow; hindgut *Allium-*derived PTSO—green). Proportion of explained variance by each PCo axis is shown.

The bacterial community of European seabass juveniles varied significantly between the two diets, considering both the most abundant bacterial ASVs (weighted UniFrac) and minority ASVs (unweighted UniFrac) (Table 3, Figure 5). Regarding both gut regions separately, significant differences appeared in both regions. In the foregut, differences between experimental diets were observed among both majority (GLMM, weighted UniFrac, diet as factor, Pseudo-F_1,84_ = 18.79, *p* = 0.001) and minority ASVs (GLMM, unweighted UniFrac, diet as factor, Pseudo-F_1,84_ = 4.99, *p* = 0.001). In the hindgut, results were similar, with differences in diet with both majority (GLMM, weighted UniFrac, diet as factor, Pseudo-F_1,92_ = 13.03, *p* = 0.001) and minority ASVs (GLMM, unweighted UniFrac, diet as factor, Pseudo-F_1,92_ = 4.89, *p* = 0.001). 

## 3. Discussion

In this study, juvenile European seabass supplemented with an *Allium*-derived organosulfur compound, such as propyl propane thiosulfonate (PTSO), produced an increased in body weight at the end of the feeding trial (89 days). This increase in growth performance was accompanied by significant changes in bacterial communities and in some bacterial groups in both foregut and hindgut, as well as a decrease in alpha diversity in PTSO supplemented fish.

The spread of antimicrobial resistance requires an urgent quest in searching for new alternatives to AGP in aquaculture. However, these new products must ensure animal welfare. Some compounds have been proposed as good AGP alternatives, such as probiotics, prebiotics, organic acids, and plant extracts [46]. Plant extracts, also known as phytobiotics, include a wide range of plant-derived products, such as essential oils, herbs, and oleoresins [17]. Phytobiotics have been proposed as good and safe AGP alternatives, capable of modulating intestinal microbiota and increasing productive parameters, while also containing anti-pathogenic and appetite stimulation properties of both terrestrial and aquatic animals [15,47]. The phytobiotics used in the animal feed come from different plant species, being the products derived from *Allium* plants the most widely used, mainly garlic (*Allium sativum*) and onion (*Allium cepa*) [36,48]. Organosulfur compounds are the most important bioactive compounds derived from *Allium*, showing antibacterial, antifungal, antiviral, anti-inflammatory, and antioxidant activities [37,38,39]. Some of the most *Allium-*derived organosulfur compounds used for animal feed include ajoene, allicin, isoalliin, methiin, propiin, propyl propane thiosulfinate (PTS), and propyl propane thiosulfonate (PTSO) [30,36]. PTSO addition has shown beneficial effects in different farm animals. In poultry, different doses of PTSO in broiler chickens improved food digestibility and growth performance and produced changes in gut microbiota [43,49,50]. Additionally, in laying hens, PTSO increased the number and the size of eggs laid and produced an increase in potentially beneficial bacteria in the intestine [27,41]. In pig industry, PTSO has shown beneficial effects in intestinal microbiota and increased growth performance in piglets and growing-finishing pigs [24,42]. The use of *Allium-*derived PTSO in aquaculture has only been studied in gilthead seabream (*Sparus aurata*) juveniles, showing potentially beneficial changes in gut microbiota and producing no changes in growth performance [51]. However, in the present study, fish supplemented with PTSO additive showed a higher body weight gain at the end of the experimental trail than control ones, supporting previous positive results of such kind of supplements in other farm animals.

Despite the few research articles on the use of PTSO in aquaculture, other *Allium-*based compounds have been used in aquafeeds in different studies and with different fish species [30]. Dietary inclusion of onion (*Allium cepa*) powder produced an increase in body weight, SGR, and immune parameters of beluga juveniles [31]. Supplementing the diet with garlic (*Allium sativum*) showed an increase in growth performance in Asian seabass (*Lates calcarifer*) [52,53]. The use of crude polysaccharides from garlic produced an increase in body weight and SGR in rainbow trout (*Onchorhynchus mykiss*) [33]. Other studies using allicin, a garlic-derived organosulfur compound, showed its benefits as growth promoter, antimicrobial agent, and feed stimulator [32]. However, the results of different studies are controversial because other studies noted the lack of effect of *Allium* extract and *Allium-*derived compounds on different fish species in aquaculture [54,55]. In fact, in a previous study using PTSO in gilthead seabream, the inclusion of this *Allium-*derived compound produced no changes in growth performance [51]. Our results with the European seabass juveniles showed no differences in body weight between control and *Allium-*derived PTSO supplemented fish along the experiment, although we found a significant increase in body weight at the end of the experimental period (after 89 days of treatment). Further studies are needed to clarify differences between phytobiotic presentation and fish species.

Our study showed a significant decrease in all the alpha diversity indices studied in the foregut and hindgut of European seabass juveniles supplemented with PTSO, except in the hindgut with Shannon diversity index. Some studies have shown that reduction in alpha diversity increased body weight in birds, and obesity in humans [56,57]. In aquaculture, results relating alpha diversity and body weight are disparate. In a previous study, [58] they found that differences in bacterial diversity did not translate into differences in body weight of largemouth bronze gudgeon (*Coreius guichenoti*). However, a study with rainbow trout (*Onchorhynchus mykiss*) suggested a correlation between an increase in body weight and an increase in bacterial diversity [59]. Previous results from our research group [51] showed no differences in body weight accompanied by an increase in alpha diversity indices in gilthead seabream juveniles supplemented with *Allium-*derived PTSO. Our results with European seabass showed an opposite trend; an increase in body weight is related with a reduction in alpha diversity. This negative association between body weight gain and bacterial diversity has been found in humans [57]. In pigs, the use of an *Allium* extract similar to the supplement we provided to our gilt-head breams reduced bacterial alpha diversity and increased body weight [42]. We cannot discard that the relation between alpha diversity and body weight could be species-dependent, so standardization in experimental setups, diets, and products might disentangle this association between body weight and alpha diversity. Moreover, we have explored the effects of PTSO in juvenile growth. Longitudinal studies along the productive life of fish would show long-term effects of PTSO supplementation in growth and microbiota of fish.

Intestinal community differed between the control and *Allium-*derived PTSO diets, either when considering majority ASVs (Weighted UniFrac) or minority ASVs (Unweighted UniFrac). These community differences are in accordance with changes in some of the majority genera of the intestinal microbiota in supplemented fish with respect to those of the control fish. The relative abundance of *Pseudomonas* increased in *Allium-*derived PTSO supplemented fish in both foregut and hindgut regions. These results could be a negative trade-off since, despite the fact that *Pseudomonas* have been described as an ubiquitous bacterial genus, some species are emergent opportunistic fish pathogens [60]. *P. anguilliseptica* is considered a fish pathogen, and it is the main causative agent of winter disease, an illness associated with several farmed fish, such as seabass, cod, and gilthead seabream [61]. Other *Pseudomonas* species such *P. aeruginosa, P. putida* or *P. fluorescens* are considered opportunistic pathogens in aquaculture [62]. However, different strains of *P. fluorescens* have shown probiotic properties in fish, improving immune system [63] or inhibiting the fish pathogenic bacteria *Vibrio anguillarum* [64]. As with many other bacteria, the pathogenic or symbiotic trait in some bacteria depends on the species and the strains. We have also found that the relative abundance of *Vibrio* in both foregut and hindgut significantly decreased in the European seabass juveniles supplemented with *Allium-*derived PTSO. *Vibrio* species are ubiquitous in marine environments, and some species are considered potentially pathogenic, causing clinical diseases as vibriosis [13,65]. *V. anguillarum*, *V. salmonicida*, *V. alginolyticus*, *V. harveyi,* or *V. parahaemolyticus* are some of the *Vibrio* species which cause the most devastating effects on marine fish [66]. Some plant extracts have demonstrated antimicrobial activity against different *Vibrio* species in aquaculture. Ginger powder and garlic powder showed antimicrobial effects against *V. harveyi* in Asian seabass [67]. The use of garlic has shown antimicrobial effects against *Vibrio* species in aquaculture [30]. A previous study [68] showed in vitro inhibitory activity of garlic (*A. sativum*) against *V. anguillarum*, *V. alginolyticus,* and *V. harveyi*. This is also true for PTSO, which has shown direct inhibition in vitro against *Vibrio*, *Pseudomonas*, *Enterobacteria*, and several Gram-positive bacteria [37,69]. Among these, *Vibrio parahaemolyticus* was the most sensitive strain against PTSO, which may explain the *Vibrio* reduction observed in this study. Further research is necessary to explore in detail different *Vibrio* and *Pseudomonas* strains in order to untangle the antagonistic relationships between bacterial species. Future studies should address the limitations of the current study, including increasing the experimental timeline to adult stage of the seabass and observing how the treatment affects the morphology of the intestinal mucosa. Peinado and colleagues [49] showed a significant increase in histometrical parameters of the small intestinal, such as villus height, width, and surface area in birds fed with 90 mg/kg of PTSO, which could explain the body weight gain due to an increased nutrient absorption via an increase in surface area.

## 4. Materials and Methods

### 4.1. Animals, Experimental Design and Fish Sampling

European seabass (*Dicentrarchus labrax*) juveniles (*n* = 780) were randomly assigned to two experimental groups (390 fish per group), consisting of triplicate tanks (400 L; 130 fish per tank). Fish were kept in a recirculating RAS D-400 water system equipped with physical and biological filters. An amount of 5–10% of the water was renewed daily, depending on the quality of water. The temperature was adjusted at 21 ± 1 °C, and a photoperiod regime of 12L/12D hours was applied. All studied fish were handled in accordance with the European Union Guidelines (Directive 2010/63/UE) for the use of laboratory animals. The Ethical Committee at the University of Granada approved the experiments, and they were endorsed by the regional government (Junta de Andalucía, Spain, ref. no. 13/04/2018/048).

The experimental diet consisted of commercial fishmeal (NUTRAPLUS, Dibaq, Spain) and the addition of the *Allium*-based product (150 mg of PTSO/kg of fishmeal) (Supplementary Table S1). After the meal homogenization, the granulated fish feed was manufactured by SPAROS I&D Nutrition in Aquaculture (Olhão, Portugal). The same diet without *Allium-*based additive was prepared as a control. SPAROS I&D Nutrition in Aquaculture checked PTSO concentration by UHPLC-ESI-MS/MS analyses, according to the method described in [70]. The *Allium-*based product used is commercialized under the trademark AquaGarlic^®^ and was supplied by DOMCA S.A. (Granada, Spain). This product is standardized in propyl propane thiosulfonate (PTSO) at a concentration of 10% and presented as a powder on inert sepiolite.

At the beginning of the experiment, fish were randomly housed in different tanks, obtaining the same initial biomass in each tank. After 2 weeks of acclimatization, fish were anesthetized with 80 mg/L of tricaine methanesulfonate (MS-222) and weighed, with average initial body weight (BW) of 3.78 ± 0.09 g. During the feeding trial (89 days), fish were fed 3–4 times per day, 6 days per week. All fish from each tank were collected, anesthetized using MS-222, and weighed on days 0, 12, 26, 42, 63 and 89. At the end of the feeding trial (89 days, according to the facilities availability and ensuring enough time for testing the experimental effect of PTSO), 20 fish per experimental tank were euthanized by an overdose of anesthesia MS-222 (400 mg/L), followed by spine severing. Fish were immediately dissected and the whole intestine was collected with sterile material. Intestines were stored in sterile 90 mm Petri dishes and transported to the laboratory, where they were kept at −80 °C until DNA extraction.

### 4.2. DNA Extraction

Intestinal pieces of approximately 100 mg were dissected from the foregut and hindgut of European seabass (*D. labrax*) juveniles using a sterile scalpel. DNA extraction was carried out following the modified salting out procedure (MSOP) proposed by [71]. An initial mechanical lysis step using a cell disrupter FastPrep FP120 (BIO 101, Thermo Savant, Irvine, CA, USA) was introduced to increase cell lysis. In summary, intestine pieces of about 100 mg were introduced in a 2 mL microcentrifuge screw cap tube filled with 100 mg of 2 mm zirconia beads and homogenized by two consecutive pulses of 30 s at speed 5 in FastPrep FP120. After this previous step, the MSOP protocol was followed. The yield of the DNA extraction was checked by 0.7% agarose gel electrophoresis. DNA concentration was measured using NanoDrop™ 2000 Spectrophotometer (Thermo Fisher Scientific, Waltham, MA, USA) and then DNA was stored at −20 °C until PCR amplification.

### 4.3. V6-V8 16S rRNA Gene Amplification and High-Throughput Sequencing

V6-V8 region of 16S rRNA gene libraries were constructed using the primer pair B969F (5′-ACGCGHNRAACCTTACC-3′) and BA1406R (5′-ACGGGCRGTGWGTRCAA-3′) [72] with Illumina adapter overhang sequences. PCR amplification was carried out using the iProof™ High-Fidelity DNA Polymerase (Bio-Rad^®^, Hercules, CA, USA) following Rabelo-Ruiz et al. [42]. The PCR products were purified and then used as template for a second PCR. In this second PCR amplification, a unique combination of two Illumina compatible barcodes were index to each sample. This unique barcoding allow that the derived sequences can be demultiplexed into their respective samples in downstream analysis. The barcodes overlapped with the sequence of the primers used in the first PCR. All PCR amplicon purifications were made using DNA Purification SPRI Magnetic Beads (Canvax^®^, Córdoba, Argentina) following the manufacturer’s instructions. PCR amplicons were checked by 1% agarose gel electrophoresis, and DNA concentrations were measured using Qubit^®^ 3.0 Fluorometer (Invitrogen™, Carlsbad, CA, USA). Afterwards, PCR amplicons were pooled in equimolar concentrations, and high-throughput sequencing was carried out with Nextera XT DNA Library Prep Kit (Illumina, San Diego, CA, USA) in paired-ends reads of 2 × 300 bp length. Sequencing was carried out in the Illumina MiSeq platform in the Institute of Parasitology and Biomedicine “López-Neyra” (Granada, Spain). 

### 4.4. Sequences Processing and Data Analysis

16S rRNA reads generated from Illumina MiSeq sequencer were analyzed using the Quantitative Insights Into Microbial Ecology (QIIME2 v2020.11; [73]) software. At the beginning, primer trimming was performed using *cutadapt* plugin [74], and pair joining was carried out using default parameters. Quality filtering was performed with a threshold of 20 Phred score. Afterward, Deblur algorithm was used for sequence clustering into ASVs (Amplicon Sequence Variants) in order to remove sequencing errors [75]. Sequences that passed quality filters were trimmed to 400 bp, giving a dataset of 10,832,912 total reads with a mean of 51,098.64 reads per sample. The fragment insertion script implemented in QIIME2 was used to align the sequences and build a bacterial phylogenetic tree based on a reference phylogenetic tree (SEPP reference Greengenes 13.8; [76]). The taxonomy was assigned based on a pretrained classifier on Greengenes 13.08 with a similarity of 99% [77]. Finally, sequences belonging to chloroplast, mitochondria or non-bacterial DNA were filtered of the ASVs table.

### 4.5. Statistics

To test the effect of different diets on body weight, we performed generalized linear mixed models (GLMM). We used mean body weight per tank as experimental unit with diet as fixed factor.

For alpha and beta diversity analyses, the ASV table was rarified at 10,000 sequencing depth per sample. Samples that did not reach this sequencing depth were excluded from subsequent analyses (this was an 11% of the samples, i.e., 22 out of a total of 200 samples). Four alpha diversity indices were calculated, i.e., Shannon diversity index [78], chao1 index [79], Faith phylogenetic diversity index [80], and OTU Richness. We used GLMM to explore the effect of diet and gut region as fixed factors in both alpha diversity indices. In alpha and beta diversity analysis, fish was used as the experimental unit.

Body weight and alpha diversity analyses were performed using STATISTICA 10.0 (StatSoft).

Differences in genera and classes abundances between control and *Allium-*derived PTSO supplemented fish were explored by means of linear discriminant analysis effect size (LEfSe) [81]. LEfSe analyses were performed on the Galaxy web platform, implemented on the public server https://huttenhower.sph.harvard.edu/galaxy/ (accessed on 4 July 2022).

Beta diversity distance matrixes were calculated using UniFrac index. Both weighted and unweighted UniFrac indices [82,83] were used for subsequent analysis. Weighted UniFrac considers the relative abundance of bacteria shared between samples, giving more importance to the most abundant bacteria. Unweighted UniFrac gives more importance to rare bacteria in the ASVs as it only considers their presence or absence irrespective of their abundance. Permutational ANOVA (PERMANOVA) was performed to test these effects on both UniFrac distance matrixes using PRIMER-7 software (PRIMER-e), implemented with PERMANOVA plugin. Principal coordinate analyses (PCoA) were performed in order to visualize the 2 first axes using EMPeror 2018.2.0 [84,85].

## 5. Conclusions

The worlds’ food demand and the ban of antibiotics as growth promoters are enhancing the appearance of new alternatives for animal production and welfare. Phytobiotics may play an important role as food additives due to their positive effect on growth performance and antimicrobial activity against certain pathogens. Although the ultimate cause has not been elucidated yet, their positive effects in animal production might be associated with change in the bacterial community composition. Our experimental supplementation of the diet of European seabass juveniles with *Allium-*derived PTSO produces a final increase in fish body weight accompanied with changes in bacterial community composition. Moreover, *Allium-*derived PTSO induced changes in some bacterial groups, especially a reduction in *Vibrio*, a potential pathogen. Our results support the positive association between diet and performance in fish. However, further research is necessary to study how this *Allium-*derived PTSO affects specific pathogenic strains and how this phytobiotic product affects the immune system and health status of fish.

## Figures and Tables

**Table 1:** General linear mixed models exploring the effects of diet in body weight in European seabass juveniles fed with a control diet or supplemented with *Allium-*derived PTSO along 89 days of experiment. Significant *p*-values are shown in bold.

	Control	*Allium*-Derived PTSO	*p*
Body Weight Day 0 (g/fish)	3.72 ± 0.05	3.84 ± 0.02	0.110
Body Weight Day 12 (g/fish)	4.64 ± 0.06	4.70 ± 0.04	0.438
Body Weight Day 26 (g/fish)	5.90 ± 0.12	5.87 ± 0.09	0.818
Body Weight Day 42 (g/fish)	8.21 ± 0.12	8.14 ± 0.08	0.640
Body Weight Day 63 (g/fish)	11.96 ± 0.09	12.25 ± 0.10	0.089
Body Weight Day 89 (g/fish)	21.14 ± 0.21	22.08 ± 0.08	0.013

**Table 2:** General linear mixed models exploring the effects of fish experimental diet (control and supplemented with *Allium*-derived PTSO) and gut region in different alpha diversity indices of bacterial community of juvenile European seabass. D.f. refers to degree of freedom. Significant *p*-values are shown in bold.

Alpha Diversity Index	Explanatory Variables	D.f	F	*p*
	Diet	1177	83.97	**<0.001**
Chao1 Index	Gut Region	1177	0.02	0.899
	Diet*Gut Region	1177	1.52	0.220
	Diet	1177	79.90	**<0.001**
Faith PD	Gut Region	1177	0.02	0.903
	Diet*Gut Region	1177	0.36	0.547
	Diet	1177	95.10	**<0.001**
OTUs Richness	Gut Region	1177	0.21	0.652
	Diet*Gut Region	1177	0.55	0.459
	Diet	1177	6.51	**0.012**
Shannon Diversity Index	Gut Region	1177	15.23	**<0.001**
	Diet*Gut Region	1177	0.96	0.330

**Table 3:** Permutational ANOVA (PERMANOVA) exploring the effects of diet, gut region, and their interaction in beta diversity indices of bacterial community of European seabass juveniles fed with control diet or supplemented with *Allium-*derived PTSO. D.f. refers to degree of freedom. Significant *p*-values are shown in bold.

β-Diversity Distance Matrix	Explanatory Variables	D.f	Pseudo-F	*p*
	Diet	1177	31.51	**0.001**
Weighted UniFrac	Gut Region	1177	14.00	**0.001**
	Diet*Gut Region	1177	0.98	0.409
	Diet	1177	8.89	**0.001**
Unweighted UniFrac	Gut Region	1177	1.05	0.325
	Diet*Gut Region	1177	0.94	0.595

## Data Availability

Raw sequences are available at the Sequence Read Archive (SRA) in Genbank (https://www.ncbi.nlm.nih.gov/sra/, accessed on 22 February 2022) under accession numbers SAMN26177506 to SAMN26177705.

## Supplementary Materials

The following supporting information can be downloaded at: https://www.mdpi.com/article/10.3390/antibiotics12010134/s1, Figure S1: Structure of PTS and PTSO; Table S1: Diet composition of the fish experimental diet.
