# Supplementary material for: Allium-Derived Compound Propyl Propane Thiosulfonate (PTSO) Reduces Vibrio Populations and Increases Body Weight of European Seabass (Dicentrarchus labrax) Juveniles
Source: Antibiotics (Basel). 2023 Jan 10;12(1):134. doi: 10.3390/antibiotics12010134 (PMC9854545; doi:10.3390/antibiotics12010134)
Supplement: Supplementary file 1 [file antibiotics-12-00134-s001.zip › antibiotics-2127301-SI.pdf]

Supplementary Materials

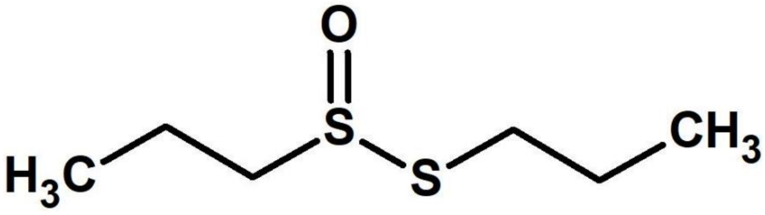

PTS

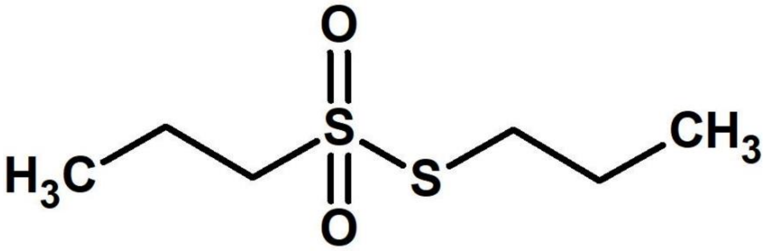

PTSO

Figure S1. Structure of PTS and PTSO.

Table S1. Diet composition of the fish experimental diet.

Nutrient composition	
Crude protein %	45.00
Crude Fat %	20.00
Cellulose %	2,8
Ash %	8.00
N.F.E. %	13,3
Moisture %	10.00
Calcium %	1,8
Phosphorus %	0,9
Gross Energy (MJ/Kg)	21,10
Digestible Energy (MJ/Kg)	17,5
Protein Digestibility %	90.00
PD/DE (gr/MJ)	23,14
PTSO (mg/Kg)	150
